# Fator de Impacto de 2,0, um Novo Recorde Histórico do ABC Cardiol – Muito Obrigado a nossa Comunidade Científica e Cardiológica

**DOI:** 10.36660/abc.20210616

**Published:** 2021-08-09

**Affiliations:** 1 Universidade de São Paulo Faculdade de Medicina Hospital das Clínicas - Instituto do Coração São PauloSP Brasil Universidade de São Paulo, Faculdade de Medicina, Hospital das Clínicas - Instituto do Coração, São Paulo, SP - Brasil; 2 Hospital do Coração São PauloSP Brasil Hospital do Coração (HCOR), São Paulo, SP – Brasil

**Keywords:** Fator de Impacto, Indicadores de Produção Científica, Fator de Impacto de Revistas, Cardiologia

A publicação científica mais tradicional da Sociedade Brasileira de Cardiologia, com 73 anos de idade, *Arquivos Brasileiros de Cardiologia* ou *ABC Cardiol*, acaba de atingir um patamar de reconhecimento internacional baseado no fator de impacto do ano 2020, que é um novo recorde na sua história.

**Figura 1:**
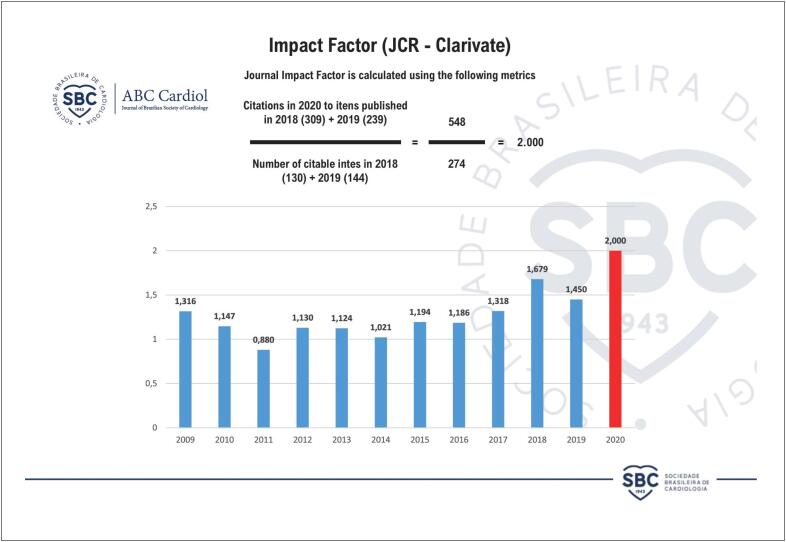
Fator de Impacto (JCR – Clarivate)

**Figura 2:**
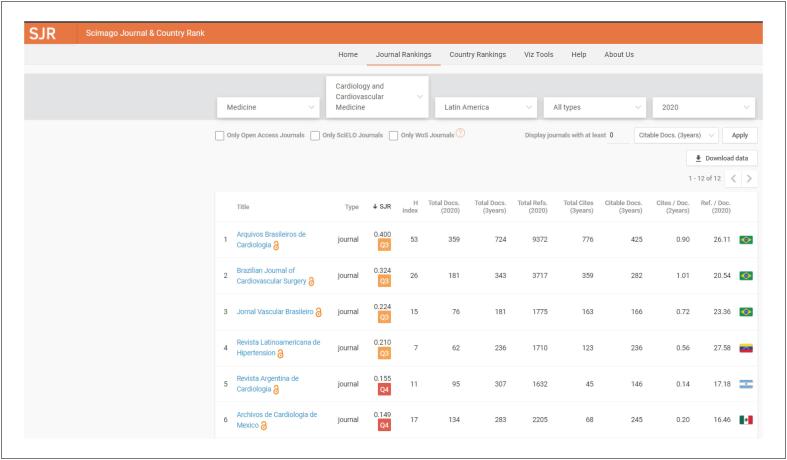
Fator de Impacto (SJR – Scimago).

O novo fator de impacto do *ABC Cardiol* é de 2,0, publicado pelo Journal of Citation Report (JCR, Clarivate) de 2020, o que significa que em média cada artigo publicado no *ABC Cardiol* teve 2 citações por outros artigos. Isto representa o impacto dos dados científicos publicados no *ABC Cardiol* na comunidade com produção científica ativa. Este índex vem subindo progressivamente nos últimos anos e o fator de impacto de 2 não é apenas um número isolado (Figura 1).^1^^–^^4^ Também de grande importância é a posição do *ABC Cardiol* como a número 1 entre as revistas de ciência cardiovascular na América Latina pelos rankings internacionais como o Scopus (Figura 2).

Este feito só foi possível graças à credibilidade do *ABC Cardiol* construída na nossa comunidade científica cardiológica no Brasil e ao redor do mundo. De fato, este é um resultado atingido graças a todos os autores, revisores e editores que continuamente tem suportado e apoiado as publicações científicas da SBC. A todos que nos tem apoiado, gostaria de deixar um “Muito Obrigado!”

Gostaria também de agradecer imensamente à SBC, nas pessoas dos seus Presidentes recentes que tem sido forças motrizes nesta jornada, Dr. Oscar Dutra, Dr. Marcelo Queiroga e Dr. Celso Amodeo. Novamente, “Muito Obrigado!”

**Figura 3:**
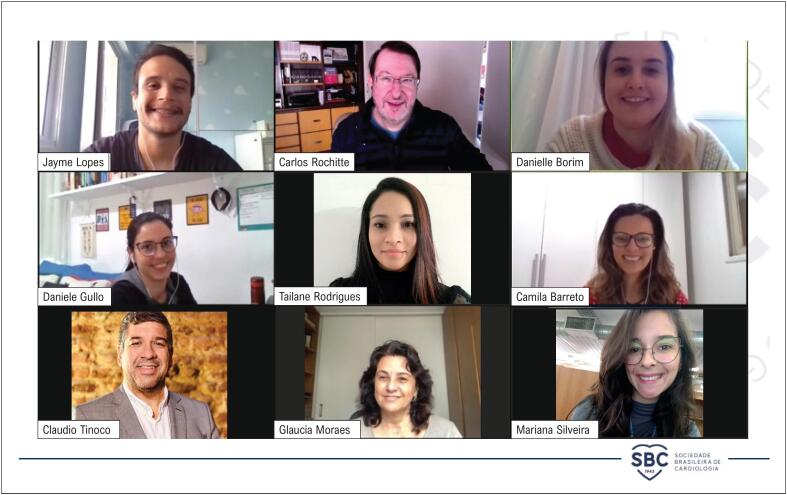
Time editorial em ação.

Ainda na SBC, nosso time de assistentes editoriais (Figura 3) são a verdadeira alma da nossa publicação e todos nós temos que mandar um grande “Muito Obrigado!”

A missão mais importante deste editorial é espalhar “Muito Obrigado!” a todos envolvidos direta ou indiretamente neste fantástico resultado que pertence a todos que são parte da nossa grande comunidade cardiológica.

Trazendo alguns dados deste novo fator de impacto, os 274 artigos publicados no *ABC Cardiol* em 2018 e 2019 receberam 548 citações em outros artigos científicos. Muitas revistas internacionais de grande prestígio citaram artigos publicados no *ABC Cardiol*, incluindo *Circulation, Journal of the American Heart Association, Journal of the American College of Cardiology, Atherosclerosis*, *Journal of Clinical Hypertension, International Journal of Molecular Sciences* e muitas outras. Os artigos citados incluem muitos artigos originais e de origem nacional e internacional, e diferentemente de anos anteriores, o fator de impacto não foi tão dependente de citações de diretrizes da SBC. Múltiplos artigos nacionais e internacionais tiveram número significativo de citações na literatura internacional.

**Figura 4:**
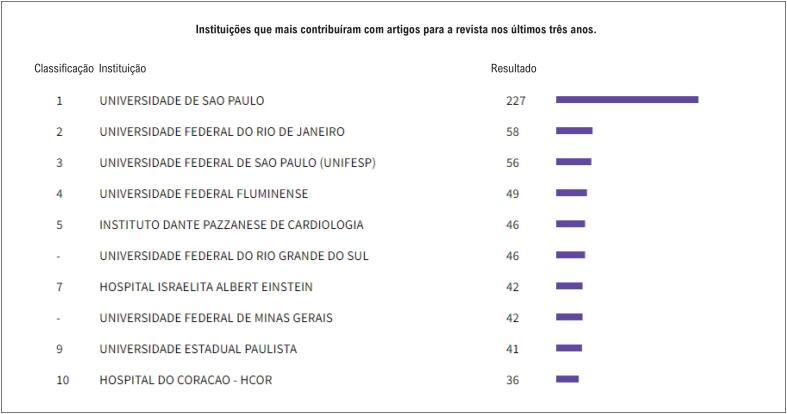
Contribuições das instituições científicas.

As contribuições dos programas de pós-graduação das universidades brasileiras foram fundamentais para nosso fator de impacto, e mais uma vez quero deixar nosso “Muito Obrigado!” As instituições nacionais que mais contribuíram para o *ABC Cardiol* nos últimos 3 anos são todas de grande tradição científica na Cardiologia nacional e são demonstradas na Figura 4.

Finalmente, a evolução qualitativa do *ABC Cardiol* permitirá expandir o escopo das publicações científicas da SBC e criar a Família de Publicações Científicas da SBC.^5^^–^^6^ Recentemente, a mais nova publicação científica da SBC foi lançada, o *ABC Heart Failure and Cardiomyopathy* e, juntamente com o *International Journal of Cardiovascular Sciences* e *ABC Cardiovascular Imaging*, vão constituir a família inicial de publicações da SBC.

O futuro do *ABC Cardiol* e das publicações científicas da SBC é brilhante e excitante. As publicações científicas da SBC com a sua credibilidade e responsabilidade^7^ constituem um veículo científico de alta qualidade a serviço da Cardiologia e comunidade científica mundial, diretamente aqui do Brasil.
