# Supplementary material for: Fator de Impacto de 2,0, um Novo Recorde Histórico do ABC Cardiol – Muito Obrigado a nossa Comunidade Científica e Cardiológica
Source: Arq Bras Cardiol. 2021 Aug 9;117(2):266–9. [Article in Portuguese] doi: 10.36660/abc.20210616 (PMC8395800; doi:10.36660/abc.20210616)
Supplement: Translation

# Translation: Impact Factor of 2.0, a New Historical Record for ABC Cardiol – Many Thanks to our Cardiology and Scientific Community

**Affiliations:** 1 São PauloSP Brazil Universidade de São Paulo, Faculdade de Medicina, Hospital das Clínicas - Instituto do Coração, São Paulo, SP - Brazil; 2 São PauloSP Brazil Hospital do Coração (HCOR), São Paulo, SP – Brazil

**Keywords:** Impact Factor, Scientific Publication Indicators, Journal Impact Factor, Cardiology

The Brazilian Society of Cardiology's most traditional scientific journal, with 73 years of history, *Arquivos Brasileiros de Cardiologia* or *ABC Cardiol*, has recently reached a new level of international recognition based on its 2020 impact factor, which is a new historical record.

*ABC Cardiol*'s new impact factor is 2.0, as published by the 2020 Journal of Citation Report (JCR, Clarivate), which means that, on average, each article published in *ABC Cardiol* was cited 2 times by other articles. This represents the impact of scientific data published in *ABC Cardiol* in the actively productive scientific community. This index has been progressively rising over the past years, and the impact factor of 2 is not merely an isolated number (Figure 1).^1^^–^^4^ It is also highly noteworthy that *ABC Cardiol* holds first place among cardiovascular science journals in Latin America in international rankings, such as Scopus (Figure 2).

**Figure 1:** Impact factor (JCR – Clarivate)

**Figure 2:** Ranking (SJR – Scimago).

This achievement was made possible by the credibility that *ABC Cardiol* has built in our scientific cardiology community in Brazil and worldwide. In fact, this result was achieved thanks to all the authors, reviewers, and editors who have continually supported the Brazilian Society of Cardiology's scientific journals. To all those who have supported us, I would like to say, “Thank you very much!”

I would also like to thank the Brazilian Society of Cardiology, represented by the recent Presidents who have been the driving forces on this journey, Dr. Oscar Dutra, Dr. Marcelo Queiroga, and Dr. Celso Amodeo. Once again, “Thank you very much!”

Also part of the Brazilian Society of Cardiology, our team of editorial assistants (Figure 3) is the true soul of our journal, and we would all like to send a big, “Thank you very much!”

**Figure 3:** Editorial team in action.

The most important mission of this editorial is to say, “Thank you very much” to all those who are directly or indirectly involved in this wonderful result, which belongs to everyone who is part of our big cardiology community.

To cite a few data related to this new impact factor, the 274 articles published in *ABC Cardiol* in 2018 and 2019 were cited 548 times by other scientific articles. Many highly prestigious international journals have cited articles published in *ABC Cardiol*, including *Circulation, Journal of the American Heart Association, Journal of the American College of Cardiology, Atherosclerosis*, *Journal of Clinical Hypertension, International Journal of Molecular Sciences*, and many others. The cited articles include many original Brazilian and international articles. This year's impact factor relied less on citations of Brazilian Society of Cardiology Guidelines than that of previous years. Multiple Brazilian and international articles were cited a significant number of times in the international literature.

The contributions of postgraduate programs and Brazilian universities have been fundamental to our impact factor, and, once more, I would like to say, “Thank you very much!” The Brazilian institutions that contributed the most papers to *ABC Cardiol* during the past 3 years all have long-standing traditions in Brazilian Cardiology, and they are displayed in Figure 4.

**Figure 4:** Contributions of scientific institutions.

Finally, the evolving quality of *ABC Cardiol* will make it possible to expand the scope of the Brazilian Society of Cardiology's scientific publications with the creation of a family of scientific journals.^5^^–^^6^ Recently, the Brazilian Society of Cardiology released its newest scientific journal, *ABC Heart Failure & Cardiomyopathy*, which, in conjunction with the *International Journal of Cardiovascular Sciences* and *ABC Cardiovascular Imaging*, will compose the Brazilian Society of Cardiology's initial family of publications.

*ABC Cardiol* and the Brazilian Society of Cardiology's scientific journals have a bright and exciting future ahead of them. The Brazilian Society of Cardiology's scientific journals, on account of their credibility and responsibility,^7^ constitute a high quality scientific vehicle at the service of Cardiology and the worldwide scientific community, right here in Brazil.
